# Compatibility of Injectable Anticoagulant Agents in Ethanol; *In Vitro* Antibiofilm Activity and Impact on Polyurethane Catheters of Enoxaparin 400 U/mL in 40% v/v Ethanol

**DOI:** 10.1371/journal.pone.0159475

**Published:** 2016-07-21

**Authors:** Damien Balestrino, Mercédès Quintana, Nicolas Charbonnel, Christiane Forestier, Claire Lartigue, Bertrand Souweine

**Affiliations:** 1 UMR CNRS 6023, Laboratoire Microorganismes: Génome et Environnement, Clermont Université, Université d'Auvergne, Clermont Ferrand, 63000, France; 2 Université d’Auvergne, Faculté de Pharmacie, Laboratoire de Bactériologie, F- 63001, Clermont-Ferrand, France; 3 Université d’Auvergne, Faculté de Pharmacie, Laboratoire de Chimie analytique et spectrométrie de masse, F- 63001, Clermont-Ferrand, France; 4 Inserm, UMR 990, IMTV, F-63005, Clermont-Ferrand, France; 5 Réanimation Médicale, Hôpital Gabriel Montpied, CHU-Clermont-Ferrand, 63000, France; National Institutes of Health, UNITED STATES

## Abstract

**Background and Objectives:**

Interdialytic lock solutions should maintain catheter patency and prevent catheter infections. We aimed to determine in which conditions injectable anticoagulant agents (IAAs) combined with ethanol are compatible and to assess the antibiofilm activity of the selected combination and its effects on dialysis catheters (DC).

**Methods:**

The solubility and compatibility of unfractionated heparin (UFH), low molecular weight heparins (LMWHs), heparinoids and fondaparinux (50 to 2,500 U/mL) in 30 to 70% ethanol were determined by visual observation. The stability of enoxaparin in ethanol and the ethanol content were assessed by high performance liquid chromatography (HPLC) and titrimetric control, respectively. The bactericidal effect was determined on 24h-old biofilms embedded in silicone-DC. The integrity of polyurethane-DC immersed in anticoagulant-ethanol was assessed by gas chromatography-mass spectrometry (GC-MS) and compared with previously published results.

**Results:**

The compatibility of IAAs and ethanol varied according to IAA type and concentration, and ethanol content. UFH in 40% ethanol was not compatible, whatever the UFH concentration used. Established limits of compatibility of enoxaparin, nadroparin, dalteparin and tinzaparin in 40% ethanol were 1350, 575, 307 and 207 U/ml, respectively, and up to 300 U/ml for danaparoid and 1 mg/mL for fondaparinux. Enoxaparin 400 U/mL in 40% ethanol (Enox/Eth) eradicated biofilm after 4 hours of exposure for *Staphylococcus epidermidis*, *Pseudomonas aeruginosa* and *Candida albicans* and after 24 hours for *Klebsiella pneumoniae* and *S*. *aureus*. Aliphatic carbonate and alcohol compounds were released by polyurethane-DC after Enox/Eth exposure, as after 40% ethanol or saline exposure. There was no significant difference between the amounts released after 30 minutes of exposure to Enox/Eth and 15 days to saline.

**Conclusions:**

A 40% ethanol solution can be combined with all IAAs but UFH. Enox/Eth was effective as an anti-biofilm agent with minor impacts on DC integrity and could be a useful interdialytic lock solution.

## Introduction

Dialysis catheters (DCs) are widely used for the provision of dialysis in end stage renal disease patients with a non-functioning arteriovenous fistula or synthetic graft [1–2]. Infection and thrombosis are two major complications associated with DC use [2–3]. DC infections represent one of the most common causes of death in chronic dialysis patients [4], with mortality rates estimated between 12 and 25% [5]. In addition, thrombosis-induced DC dysfunction is a frequent reason for DC removal or replacement [6]. At the end of each dialysis session, unfractionated heparin (UFH) is currently instilled into DC lumens and left in between the dialysis sessions to maintain catheter patency. UFH is the injectable anticoagulant agent (IAA) generally used for this purpose but other IAAs such as low molecular weight heparins (LMWHs), pentasaccharide Factor Xa inhibitor (fondaparinux) and heparinoids could also be envisaged as lock solutions. In particular, LMWHs seem to generate less adverse reactions in the long term [7]. In vitro studies assessing the antibiofilm activity of UFH have yielded conflicting results [8–9]. UFH at low concentrations (< 1,000 U/mL) have been shown to stimulate *Staphylococcus aureus* biofilm formation [8,10]. Higher inhibitory effects are obtained when chelating agents are used in combination with anti-microbial agents such as antibiotics [11]. However, the widespread use of solutions containing a high concentration of antibiotics as lock solutions raises concerns in clinical practice because of side effects [12] and the risk for development of antimicrobial-resistant organisms [13–14]. Ethanol is an antiseptic that exerts bactericidal and fungicidal activity against a broad range of microorganisms. It acts by non-specific protein denaturation and thus is less likely to promote antimicrobial resistance. Several randomized control studies have assessed the efficacy of ethanol locks in preventing catheter infections and have yielded conflicting results [15–19]. These discrepancies may be related to differences in study design, case definition, case mix population, type of catheter, ethanol lock concentration and dwell time. Of these studies, only one focused on chronic hemodialysis patients with long-term tunneled catheters. The authors reported that locking catheter between dialysis sessions once a week with 70% (v/v) ethanol and twice with UFH was associated with a 67% decrease in catheter-related bloodstream infections as compared to standard interdialytic UFH lock thrice a week [17]. However, prolonged and repeated instillation with 70% (v/v) ethanol concentration may be associated with catheter damage and obstruction requiring catheter withdrawal [18]. Experimental data suggest that there is no need to use a 70% (v/v) ethanol solution to eradicate sessile microorganisms and that ethanol at lower concentrations, about 40% (v/v), exert antibiofilm effects [20–23]. Our preliminary studies on DCs reported that immersion in 40% ethanol has only a marginal impact on catheter integrity [24,25]. However, ethanol has no anticoagulant properties and should not be mixed with UFH because of precipitation [26]. UFH is a heterogeneous mixture of polysaccharides with variable molecular weight and whether ethanol causes precipitation of fractionated heparin, pentasaccharide Factor Xa inhibitor, and heparinoids remains unknown. These treatments are increasingly used for anticoagulation during dialysis sessions [7,27,28] and could be an appropriate alternative for inter-dialytic lock solutions [29]. The aim of the study was to assess the compatibility of IAAs in ethanol and to determine the anti-biofilm activity of the optimal combination against several microorganisms commonly involved in catheter-related infections and its effect on the integrity of polyurethane DCs.

## Materials and Methods

### Ethic Statement

Whole blood (WB) samples were obtained from healthy blood donors at the local French blood agency (Etablissement Français du Sang [EFS], Saint-Etienne, France). In France, the use of blood samples from donors for research purpose is controlled by the State. The Law indicates that blood donation requires the systematic information of the volunteers (article R.1221-5 of the Public Health Code, 01/12/2009 and 06/11/2006 decrees) and that written informed consents must be obtained by EFS from all donors whose samples are involved in research studies. The EFS controls the storage of the samples and their use in any study, in a legal and ethical framework, and there is no requirement for approval by a local Ethical Committee

### Chemicals, Reagents and Materials

Mixing solutions were prepared using various commercially available injectable anticoagulant agents (IAAs): unfractionated heparin (UFH), heparin sodium (Choay, 5,000 U/mL, Sanofi-Aventis, France); low molecular weight heparins (LMWHs), enoxaparin sodium (Enox), (Lovenox®, 10,000 U/mL, Sanofi-Aventis, France), tinzaparin sodium (Innohep®, 20,000 U/mL, Leo Pharma, France), nadroparin calcium (Fraxiparine®, 9,500 U/mL, GlaxoSmithKline, France), dalteparin sodium (Fragmine®, 10,000 U/mL, Pfizer, France); and a heparinoid, danaparoid sodium (Orgaran®, 1,250 U/mL, MSD, France), pentasaccharide Factor Xa inhibitor, sodium fondaparinux (Arixtra®, 12.5 mg/mL, GlaxoSmithKline, France). In this study the wording IAA refers to UFH, LMWHs, fondaparinux and danaparoid sodium. Mixing solutions were prepared in 5 mL glass hemolysis tubes, combining either a IAA fixed concentration and increased concentrations of ethanol and prepared with absolute ethanol in 0.9% sodium chloride solution (saline solution), or increased concentrations of IAA between 50 and 1,000 U/mL (and in some cases up to 2,500 U/mL) and a fixed ethanol content. All ethanol contents in the work are expressed in % (v/v). The ethanol content in the mixing solutions ranged between 30 and 70% in saline or in some experiments up to 95% ethanol. Absolute ethanol (purity more than 99.9%) for high performance liquid chromatography (HPLC) was obtained from Carlo Erba (Peypin, France), HPLC quality methanol was from Acros-Organics (Van Overbeek, Belgium) and 0.9% sodium chloride from Aguettant (Lyon, France). Ethyl acetate, used as solvent for extraction, was of analytical grade (Carlo Erba, Peypin, France), and cyclododecanol, used as GC-MS internal standard (IS), was from Sigma Aldrich (Saint Quentin Fallavier, France).

*Staphylococcus epidermidis* CIP 68.21, *S*. *aureus* CIP 65.25 (methicillin resistant), *P*. *aeruginosa* ATCC 27853, *Klebsiella pneumoniae* LM21 and *C*. *albicans* SC5314 were selected for the microbial study. Bacterial strains were grown in lysogeny broth and in minimal medium (M63B1) and the fungal species in 0.67% yeast nitrogen base (YNB, Difco) supplemented with 0.4% glucose. The organisms were maintained at −70°C in their respective medium with 15% glycerol, and on each occasion the biofilm was established from the original stock.

The catheters tested for microbial analyses were segments (each 1 cm long) of sterile silicone dialysis catheters (S-DCs) (DualCath®, Medcomp, Harleyville, PA, USA, and Hemotech, Ramonville, France), which are easier to cut and fix onto the glass slide of the microfermentors than polyurethane catheters. For chemical analysis, the catheters were 60 cm long unstuffed tunneled polyurethane catheters (PU-DCs) (Carbothane®, 85A, Medcomp, Harleyville, PA, USA) with no outside paint marks.

### Visual Observations

A single blinded observer assessed the IAA in ethanol for color and clarity under normal lighting in a colorless glass vial held against white and black backgrounds. The appearance of any precipitate including cloudiness, film deposit and droplets was recorded after 1 min and 1, 24, 48 and 72 h of incubation at room temperature and at 37°C. Visual observation was used to determine the limits of solubility of IAA in ethanol at various IAA concentrations and in various ethanol contents. Observed solubility data of IAA in ethanol were plotted on a graph between IAA concentration (x) and ethanol content in % (y) for 72 h at room temperature and at 37°C. The relationship between the particular values of x corresponding to the IAA limit of solubility at a particular level of ethanol content y was subsequently established.

### Ethanol Assay

Ethanol content was determined by a titrimetric assay used to measure blood alcohol levels [30] with samples of IAA in ethanol previously diluted at 1:100. Ethanol stability was assayed on Enox at 400 U/mL (Enox400) in ethanol. A control assay without enoxaparin was carried out at the same time to take into account possible errors resulting from oxidation of Enox.

### HPLC-ELSD Determination of Enoxaparin

The HPLC-ELSD method (S1 Text) was used to measure enoxaparin concentrations in Enox400/ethanol mixtures at different levels of ethanol content (range 30 to 70%). Enoxaparin concentrations in Enox400 without ethanol were determined at the same time. The 95% confidence interval was determined by repetitive analysis (n = 6) of Enox400 samples in saline.

### Definitions

**Solubility**—IAA solubility in ethanol was defined at a given temperature as the absence in visual observations of any precipitate, including cloudiness, film deposit and droplets.

**Compatibility—**The compatibility of IAA in ethanol was defined when IAA solubility in ethanol was observed after 1 min, 1, 24, 48 and 72 h of incubation at room temperature and at 37°C.

**Limit of solubility**—The limit of solubility was defined at a given temperature as the highest IAA concentration soluble in ethanol at a particular level of ethanol content.

**Limit of compatibility**—The limit of compatibility was defined as the highest concentration of IAA soluble in ethanol at a particular level of ethanol content at room temperature and at 37°C.

**Ethanol stability**—The stability of ethanol in Enox/ethanol solutions was defined when the measured ethanol content was within 100.0 ± 2.0% of the theoretical value.

**Enox stability**—As requested in industry quality guidelines [31], a 95% two-sided confidence interval was used to verify enoxaparin stability. Enox400 stability in mixing solutions was defined when the measured concentration of Enox was within the range of the mean calculated concentration ± 2 SD ([387.2–415.2]).

### Silicone Catheter Biofilm Formation

Biofilm was formed on an S-DC in 60-ml aerated microfermentors as described by Ghigo [32]. Sterile S-DCs were fixed onto the internal removable glass slide of the microfermentors. Strains from the frozen stocks were cultivated in M63B1-0.4% Glu or YNB-0.4% Glu medium overnight. An inoculum of 10^9^ bacilli, 10^8^ cocci or 10^7^ *C*. *albicans* cells was used to inoculate microfermentors containing the silicone segments. Continuous flow of 100 mL/h of either M63B1-0.4% Glu medium (bacterial strains) or YNB-0.4% Glu (yeast) and constant aeration with sterile pressed air (0.3 bar) were used to obtain continuous flow-through culture conditions. Our experimental model received a high input of fresh medium to avoid significant planktonic growth. After 24 h of incubation, the S-DC segments were removed from the incubator and separated from the device. The biofilms formed on the S-DC segments were resuspended in 5 ml M63B1 minimal or YNB medium by sonication and vortexing. Serial dilutions of the resulting suspensions were performed and plated onto appropriate agar plates to determine the number of viable cells [Colony Forming Unit (CFU)] after overnight incubation at 37°C. The bacteria count was expressed as a decimal logarithm (log10). The limit of detection in our experimental conditions was 1.6 log10 (40 CFU) per KT segment.

### Microbial Treatment Protocol

After incubation, the S-DC segments harboring 24-hour biofilm were removed. Each segment was carefully rinsed in 1 mL of saline, and then placed in a tube containing 1 mL of the different lock solutions: (i) ethanol at 40%, (ii) sodium enoxaparin 400 U/mL, (iii) Enox/Eth mixing solution and (iv) 0.9% sodium chloride as control. For every organism, the experiments were repeated in triplicate or quadruplicate, and during each treatment assay S-DC segments were exposed to the different solutions for 4, 24 and 48 h at 37°C. Subsequently, the S-DC segments were removed, rinsed once with saline and the number of adherent viable microorganisms (CFU) was determined as described above. In addition, the biofilm biomass was determined in triplicate for each strain before treatment.

### Chemical Analysis of Polyurethane Catheters

Each Carbothane® PU-DC was entirely immersed in Enox/Eth and kept at 37°C for 30 minutes, 4 hours or 15 days using the protocol described elsewhere [25]. Three catheters were used with each immersion condition and GC-MS analysis in electron impact mode was performed as previously described on the immersion media using dodecanol as internal standard and extraction with ethyl acetate [25].

GC-MS qualitative analysis was performed in full scan mode to determine the chromatographic profile and establish structures of migrating components observed following immersion of Carbothane® PU-DC in Enox/Eth for 15 days. Mass spectra of compounds separated by gas chromatography were recorded and compared with those of previously identified compounds in immersion solutions (40% ethanol and control saline) of PU-DC [25]. All characterized structures were related with the aliphatic polycarbonate-polyurethane structure of the Carbothane® polymer. Quantitative GC-MS analysis was carried out by selected ion monitoring mode (using characteristic ions in mass spectra at m/z 82, 83, 89, 101 and 139) on ethyl acetate extracts [25]. Reconstructed ion chromatograms (sum of characteristic ions) were recorded on the ethyl acetate extracts of Enox/Eth immersion solutions and compared with those obtained in saline as control and in 40% ethanol alone. The areas of all GC-MS peaks were summed and the mean ratios (sum of all peaks areas/internal standard area) were calculated for comparative quantitative analysis.

### Protein Precipitation and Hemolysis Assessments

Protein precipitation and hemolysis capacity of Enox/Eth was assessed using whole blood (WB) samples obtained from five healthy blood donors at the local French blood agency (Etablissement Français du Sang [EFS], Saint-Etienne). Blood donation requires the systematic information of the volunteers (article R.1221-5 of the Public Health Code, 01/12/2009 and 06/11/2006 decrees) and written informed consents were obtained by EFS from all donors involved in our study. WB samples (0.5 and 1 ml) were diluted with 4.5 or 4 mL, respectively, of the solution to be tested, i.e. enoxaparin 400, Enoxaparin 400 in 40% ethanol mixing solution or saline (control), thus the final suspensions contained 10% and 20% of WB. After 20 min of incubation at room temperature (20°C), the samples were centrifuged at 20°C and 4,000 r.p.m. for 10 min (Eppendorf centrifuge 5810R). If there were visible signs of precipitation, serum and precipitate were transferred into polypropylene tubes and centrifuged at 20°C and 10,800 r.p.m. for 10 min (Abbott Laboratories centrifuge 3530). The supernatant was removed and the precipitate was dissolved in 0.9% sodium chloride. Albumin was assayed with a Vista® analyzer (Siemens Healthcare Diagnostics, Saint-Denis, France). The tests were performed in triplicate.

To assess hemolysis capacity, WB samples were diluted 1:200 in saline containing enoxaparin 400 or Enox/Eth, or neither (control). The dilute WB samples were incubated at room temperature for 30 min and then centrifuged at 400g/5 min/25°C. An aliquot of supernatant from each centrifuged sample and absorbance (Abs) was read at 540 nm.

### Statistical Analysis

The level of significance was set at *P* <0.05 for all the tests. The HPLC data (means ± SD) of enoxaparin determined in various ethanol contents and in saline for stability studies were compared using Statview software (SAS Institute, Cary, NC, USA) and the Wilcoxon and Mann-Whitney U tests.

Concordance between the solubility and stability tests was assessed in Enox/ethanol solutions at a fixed Enox concentration of 400 U/mL (Enox400) and 40% ethanol using the Mac Nemar test.

The microbial data expressed as means ± SD decimal logarithm (log10) of CFU were compared using the Mann-Whitney U test.

The Enox/Eth effect on PU-DC was carried out using the mean ratios of released compounds determined by GC-MS on three catheters for each immersion condition (solvent and time of contact). The Wilcoxon test was used to compare repeated measurements over time. Differences between groups (Enox/Eth, 40% ethanol and saline) were compared using the Mann-Whitney U test. Data were analyzed using Statview 5.0 software (SAS Institute, Cary, NC, USA).

## Results

### Solubility Assessed by Visual Observations

In UFH/ethanol solutions, for an ethanol content ≥ 40%, no compatibility was observed whatever the UFH concentration between 50 and 2,500 U/ml used. The compatibility of UFH in ethanol was observed using ethanol content at 30% and UFH concentrations from 50 to 2,500 U/ml, or ethanol at 35% and UFH concentration ≤ 100 U/ml. The results of the visual observations of UFH in ethanol are given in S1 Table.

**Fig 1:**
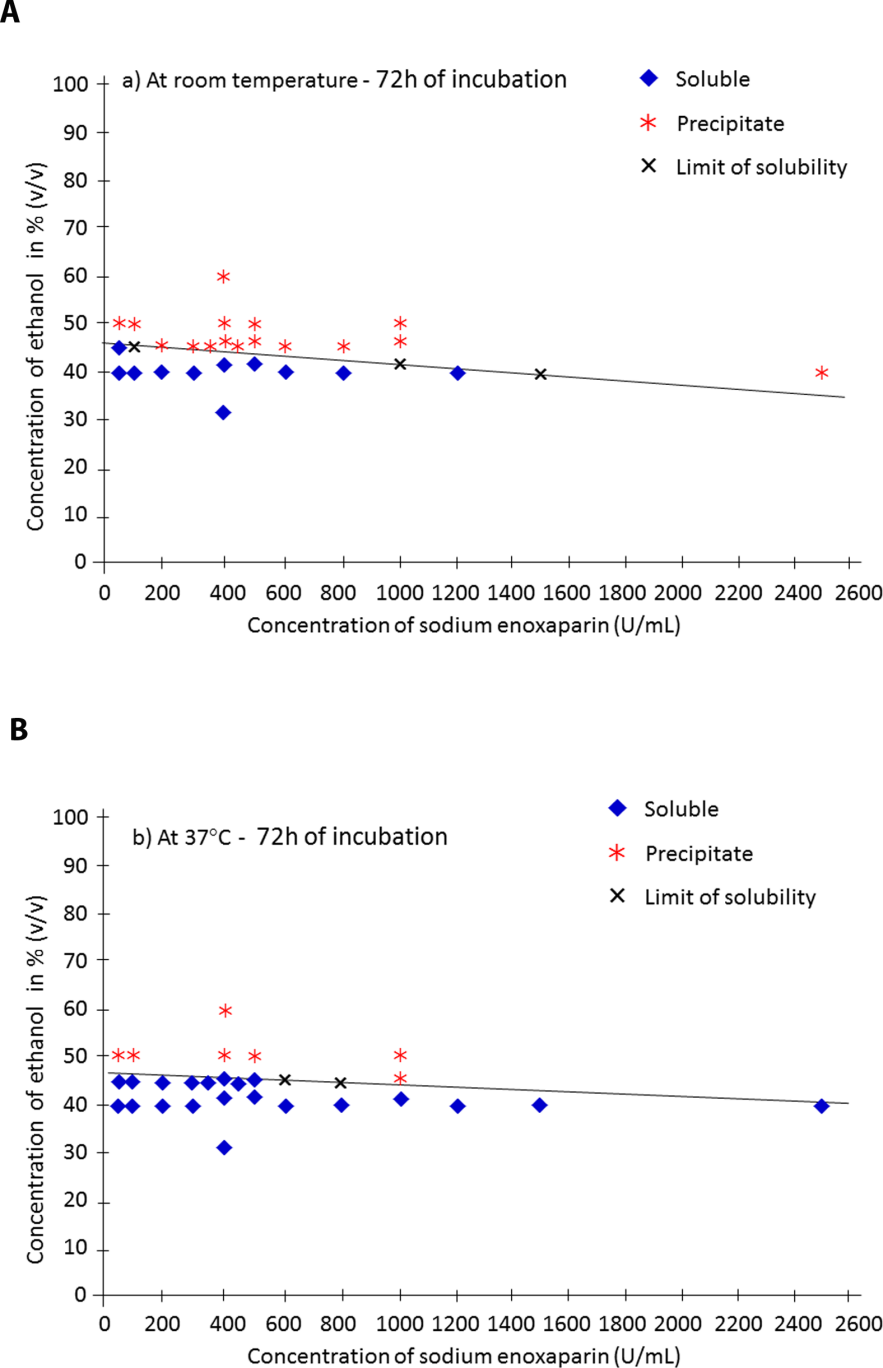
Visual determination of Enox solubility (in U/mL) according to ethanol content (in %) and linear trend curves of the limits of solubility following 72h of mixing a) at room temperature and b) at 37°C.

In Enox/ethanol solutions for an ethanol content of 40%, compatibility was observed at all Enox concentrations from 50 to 1,200 U/ml. For a 45% ethanol content, compatibility was observed when Enox concentration was ≤ 100 U/ml. When ethanol content was ≥ 50%, no solubility was observed whatever the Enox concentration ≥ 50 U/ml. Precipitates were observed immediately (1 min) and were more extensive at room temperature than at 37°C. At longer contact times, decantation occurred and a film deposit or insoluble fine droplets appeared at the bottom of the tubes, after which the supernatant solution gradually became clear again. The results of visual observations of Enox in ethanol at room temperature and at 37°C are given in Table 1 and S2 Table. The linear trend curves of the limits of solubility of Enox in ethanol are shown in Fig 1A and 1B.

**Table 1:** Testing grid for the visual determination of enoxaparin precipitation in ethanol. Influence of relative concentrations, time of contact and temperature.

Ethanol	Enoxaparin	Room temperature					37°C				
(%, v/v)	(U/mL)	1min	1h	24h	48h	72h	1min	1h	24h	48h	72h
40	50	0	0	0	0	0	0	0	0	0	0
	100	0	0	0	0	0	0	0	0	0	0
	200	0	0	0	0	0	0	0	0	0	0
	300	0	0	0	0	0	0	0	0	0	0
	400	0	0	0	0	0	0	0	0	0	0
	500	0	0	0	0	0	0	0	0	0	0
	600	0	0	0	0	0	0	0	0	0	0
	800	0	0	0	0	0	0	0	0	0	0
	1000	0	0	0	0	0	0	0	0	0	0
	1200	0	0	0	0	0	0	0	0	0	0
	1500	+	+	+	+	+	0	0	0	0	0
	2500	+	+	+	+	+	0	0	0	0	0
45	50	0	0	0	0	0	0	0	0	0	0
	100	0	0	0	0	0	0	0	0	0	0
	200	+	+	+	+	+	0	0	0	0	0
	300	+	+	+	+	+	0	0	0	0	0
	400	+	+	+	+	+	0	0	0	0	0
	500	+	+	+	+	+	0	0	0	0	0
	600	+	+	+	+	+	0	0	0	0	0
	800	+	+	+	+	+	+	+	+	+	+
	1000	+	+	+	+	+	+	+	+	+	+
50	50	+	+	+	+	+	+	+	+	+	+
	100	+	+	+	+	+	+	+	+	+	+
	400	+	+	+	+	+	+	+	+	+	+
	500	+	+	+	+	+	+	+	+	+	+
	1000	+	+	+	+	+	+	+	+	+	+

0, absence of precipitates including cloudiness, film deposit and droplets

+, presence of precipitates including cloudiness, film deposit and droplets

**Fig 2:**
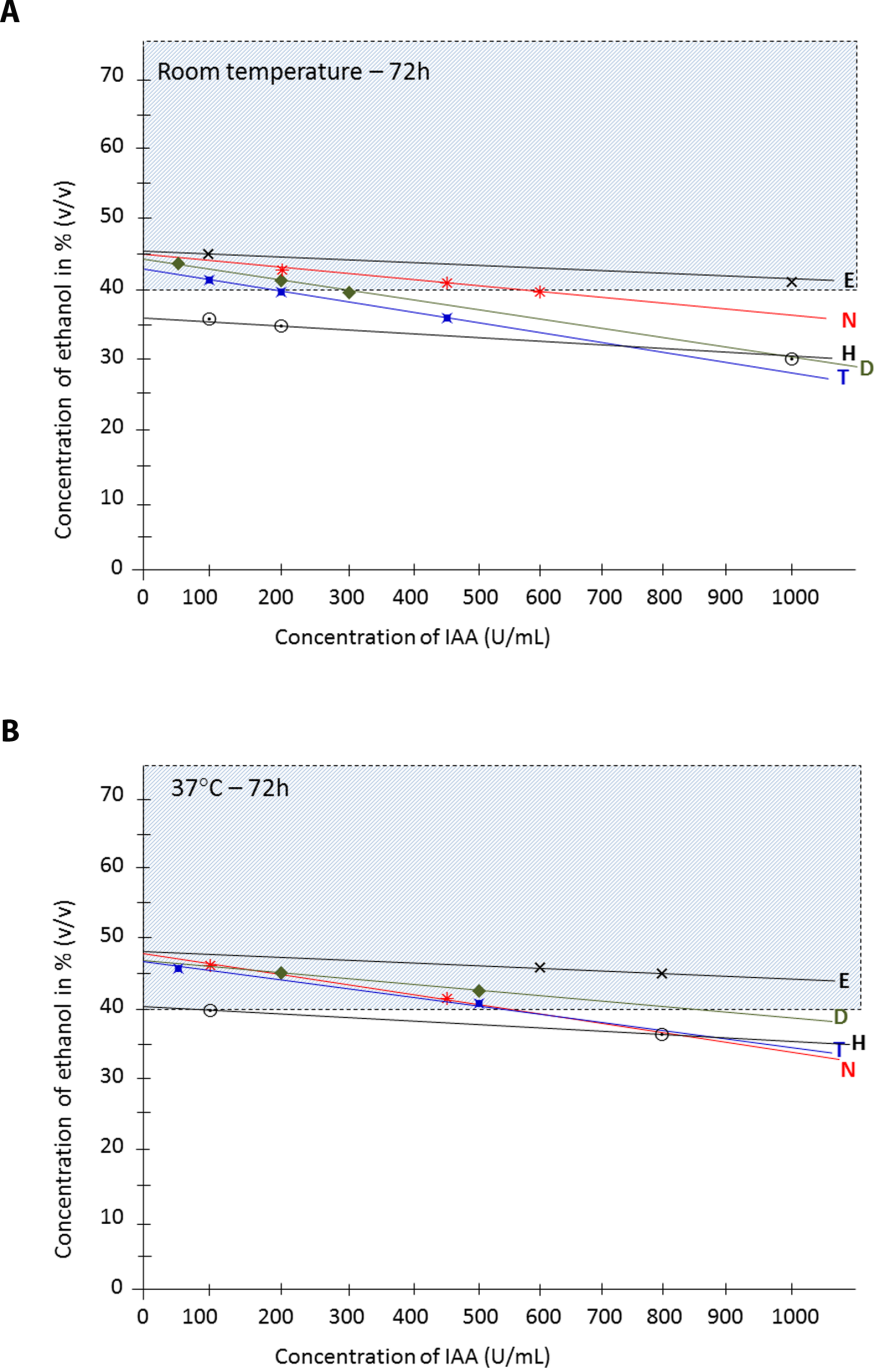
Linear trend curves of the limits of solubility of UFH (heparin: H) and LMWHs (Enox: E, tinzaparin: T, nadroparin: N and dalteparin: D) in ethanol a) at room temperature and b) at 37°C.

Similarly, the limits of solubility of tinzaparin, nadroparin, dalteparin and UFH in ethanol based on visual observations at room temperature and at 37°C are shown in Fig 2A and 2B. Since all heparins (UFH and LMWHs) in ethanol without any precipitation at room temperature were precipitate-free at 37°C, their limits of solubility in ethanol at room temperature were used to define the limits of compatibility. Equations used to determine the limits of compatibility of heparins in ethanol are given in Table 2. As estimated by equations, the limits of compatibility of heparins in 40% ethanol were 1,350 U/ml for Enox, 575 U/ml for nadroparin, 307 U/ml for dalteparin, and 207 U/ml for tinzaparin.

**Table 2:** Equations estimating the limit of compatibility of IAAs in ethanol.

Injectable anticoagulant agents / ethanol lock solutions	Formula
Unfractionated heparin / ethanol	y = -0.005x + 36.5
Enoxaparin / ethanol	y = -0.004x + 45.4
Nadroparin / ethanol	y = -0.008x + 44.6
Dalteparin / ethanol	y = -0.014x + 44.3
Tinzaparin / ethanol	y = -0.015x + 43.1

x, injectable anticoagulant agents; y, ethanol content in % (y)

In danaparoid/ethanol solutions, for 40% ethanol, compatibility was observed when danaparoid concentration was up to 300 U/ml. For 45% ethanol, compatibility was observed at danaparoid concentrations up to 200 U/ml. No compatibility was observed for danaparoid concentrations of 100 U/ml in 50% ethanol (S3 Table).

In fondaparinux/ethanol solutions, for ethanol content up to 60%, compatibility was observed at all fondaparinux concentrations between 0.1 and 1 mg/mL (S4 Table).

### Stability of Enox400/Ethanol Solutions

Measured ethanol contents in Enox400/ethanol solutions at ethanol contents of 40%, 45%, and 50%, were within 100.0 ± 2.0% of the theoretical values, showing that ethanol was stable in these combinations (S5 Table).

In Enox400/ethanol solutions, Enox400 was stable when ethanol content was ≤ 40% (Table 3). There was a significant decrease between theoretical and calculated Enox concentrations when the ethanol content was ≥ 45%. The higher the ethanol content increased the greater the stability of Enox400 decreased (Table 3 and S5 Table). For an ethanol content of 70%, Enox concentration in the clear supernatant obtained after centrifugation was below the limit of detection (LOD) of the method (< 25 U/mL in diluted samples) (S1 Fig). The HPLC analysis of droplets formed at the bottom of the tubes after centrifugation evidenced the presence of Enox in the droplets (data not shown). For Enox400/ethanol solutions there was a concordance between compatibility and stability. HPLC determination of enoxaparin concentration in Enox400/ethanol solutions at various ethanol contents (30% to 70%) indicated that Enox concentrations were unchanged in Enox/ethanol solutions without precipitates, whereas Enox concentrations decreased in mixing solutions containing precipitates (*P* = 1).

**Table 3:** HPLC-ELSD determination of enoxaparin concentrations of Enox400 in 30% to 70% ethanol at room temperature.

% EtOH	Calculated enoxaparin concentration	Mean value	Coefficient of variation	Recovery	*P*
(v/v)	(U/mL)	(U/mL) ± SD	(%)	(%)	
30%	396.5	396.8 ± 2.1	0.5	99.2	0.4386
	394.9				
	399.1				
40%	403.8	405.5 ± 2.5	0.6	101.4	0.4386
	408.4				
	404.4				
45%	381.3	381.9 ± 0.8	0.2	95.5	0.0201
	382.9				
	381.6				
50%	333.5	332.1 ± 1.3	0.4	83.0	0.0201
	331.1				
	331.6				
60%	221.8	226.6 ± 4.4	1,9	56.6	0.0201
	227.6				
	230.4				
70%	< LOD	-	-	-	-
	< LOD				
	< LOD				

LOD: limit of detection

*P* value in the 95% confidence interval

### Anti-Biofilm Activities of Enoxaparin/Ethanol-Lock Solution

**Fig 3:**
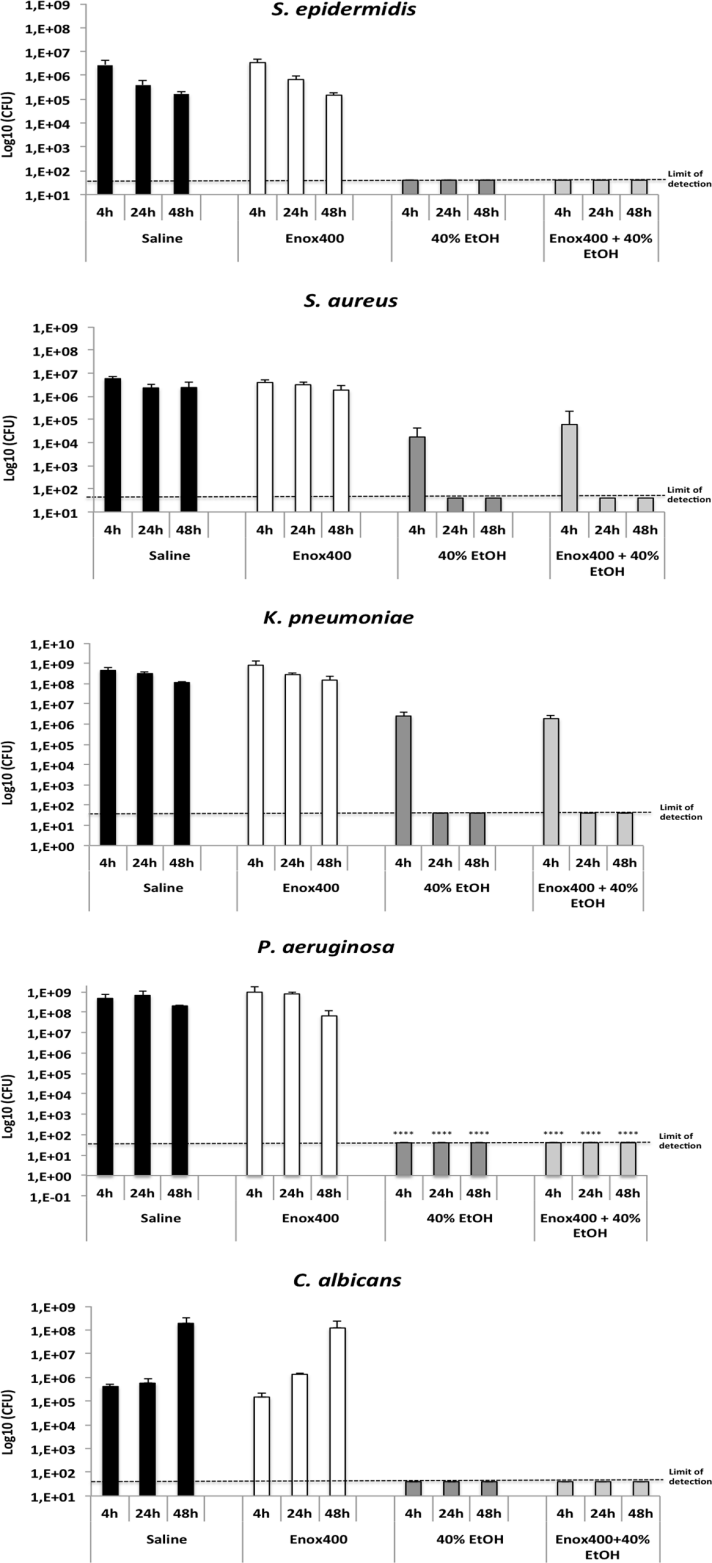
Effect of 40% Ethanol, Enoxaparin 400 U, Enoxaparin 400–40% Ethanol and saline control on microbial colony counts of different organisms in 24-hour old biofilms. Each bar represents mean CFU per centimeter of catheter at different time points along with respective standard errors of the means. The y axis is on a logarithmic scale. The limit of detection is 40 CFU and * indicates P<0.05 compared to the control at the same time.

Results of the anti-biofilm activity of the Enox400/Ethanol 40% solution are presented in Fig 3, 3A, 3B and 3C. The mean baseline counts of *S*. *epidermidis*, *S*. *aureus*, *K*. *pneumoniae*, *P*. *aeruginosa* and *C*. *albicans* adhered to catheters were 7.4× 10^6^ cfu, 4 .6× 10^6^ cfu, 7.2× 10^8^ cfu, 3.2 × 10^9^ cfu and 1.8× 10^6^ cfu, respectively. Whatever the microorganism, there were no differences in the number of viable cells after 4, 24 and 48 hours between enoxaparin 400U/mL and control saline. In contrast, after a 4-hour exposure to 40% ethanol or Enox/Eth, there was a decrease in biofilm mass, whatever the microorganism tested as compared to saline. Microorganisms were eradicated after a 4-hour exposure to both 40% ethanol and Enox/Eth for *P*. *aeruginosa*, *S*. *epidermidis*, and *C*. *albicans* biofilms, and after 24 hours for *S*. *aureus* and *K*. *pneumoniae* biofilms. Whatever the sessile microorganisms tested, eradication was achieved with both 40% ethanol and Enox/Eth after 24, 48, and 72h of treatment. There were no differences in the number of viable microorganisms between treatment with 40% ethanol and Enox/Eth whatever the microorganisms and treatment duration.

### Integrity of PUR Catheters Immersed in Enoxaparin/Ethanol-Lock Solution

**Fig 4:**
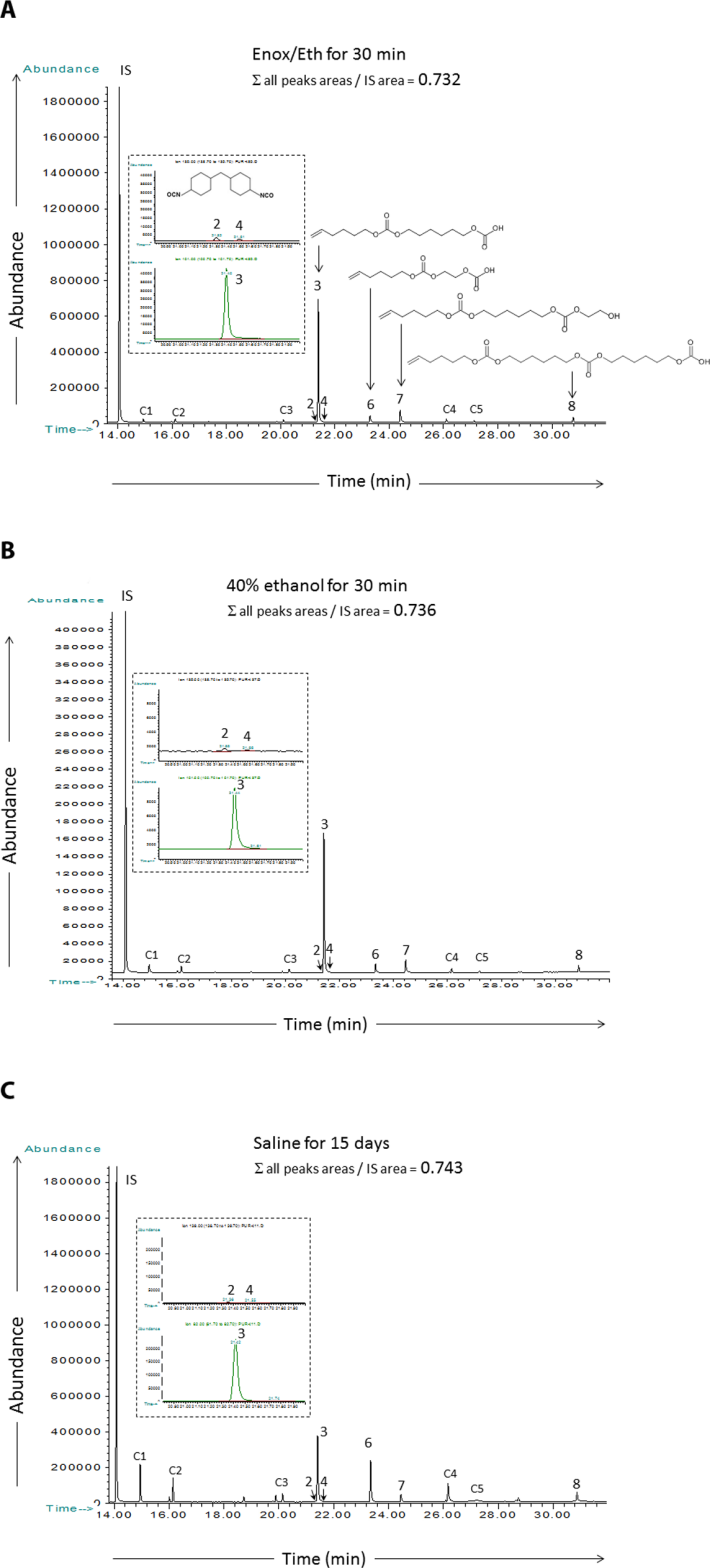
**GC-MS Total ion current profiles of ethyl acetate extracts obtained from immersion solutions in contact with Carbothane® PU-DC at 37°C:** (A) Enox/Eth immersion solution for 30 min. (B) 40% ethanol solution for 30 min. (C) Reference saline solution for 15 days. Proposed structures of release compounds are given in a) 2 and 4 are H_12_MDI isomers, 3, 6, 7 and 8 are main aliphatic alcohol and carbonates structures. C1 to C5 are minor aliphatic alcohol and carbonate structures.

GC-MS profiles for qualitative analysis of the compounds, released following immersion of Carbothane® PU-DC in Enox/Eth, were identical to those obtained following immersion in 40% ethanol and control saline solution (Fig 4A, 4B and 4C). Mass spectra analysis of chromatographic peaks showed that the release compounds were the same as those previously observed following immersion in 40% ethanol alone, and no new compound was detected. All the characterized structures were related to the complex polycarbonate-polyurethane structure of the Carbothane® polymer whose synthesis constituents are aliphatic polycarbonates and isocyanates. Major released compounds were aliphatic carbonate and alcohol structures (Fig 4A). Minor compounds (< 2% of the sum of all the compounds released) were related to the aliphatic 4,4’-dicyclohexylmethane diisocyanate isomer (H_12_MDI) structure of the polymer (Fig 4A).

Table 4 shows the mean ratios and standard deviations (n = 3) of the sum of the compounds released observed following immersion of Carbothane® PU-DC in Enox/Eth and those previously measured in ethanol at 40% [25]. There was no significant difference in the amounts of compounds released between Enox/Eth and 40% ethanol after 30 min, 4 hours and 15 days (*P* = 0.8273, *P* = 0.2752, and *P* = 0.1266 respectively). The mean value of compounds released after 30 minutes of exposure to Enox/Eth was lower than this observed after 15 days of exposure to saline: 0.694 ± 0.049 vs 0.837 ± 0.127 (*P* = 0.0492), respectively. In contrast, as compared to 15 days exposure to saline, the mean values of compounds released after 4 hours and 15 days of exposure to Enox/Eth were higher: 1.920 ± 0.447 (*P* = 0.492), and 4.775 ± 0.619 (*P* = 0.492), respectively.

**Table 4:** Mean ratios ± standard deviations (SD) of the sum of released products observed following immersion of Carbothane ®catheters (n = 3) in the mixing solutions of Enox/Eth, 40% ethanol and saline for storage times of 30 min, 4 hours and 15 days.

Storage time	Sum of released products		
	Mean ratio ± SD (n = 3)		
	Enox/Eth	40% ethanol	Saline
30 min	0.694 ± 0.049	0.677 ± 0.068	0.038 ± 0.007
4 h	1.920 ± 0.447	2.443 ± 0.289	0.151 ± 0.048
15 days	4.775 ± 0.619	6.122 ± 0.730	0.837 ± 0.127

### Protein Precipitation and Hemolysis Assessments

Protein precipitation was visually detected in all WB suspensions performed in enoxaparin 400 and in Enox/Eth, but not when WB was mixed with saline alone. The mean values of albumin in the precipitates of the suspensions at 10% and 20% WB ratios were 14.2 ± 7.1 and 11.0 ± 3.4 mg/L for Enoxaparin and 26.9 ± 13.8 and 103.7 ± 43.3 for Enox/Eth, respectively (Table 5). The hemolysis capacities of the different solutions are given in Table 6.

**Table 5:** Results of *in vitro* albumin precipitation test (mg/L).

	Test solutions ^(1)^	Saline (control)		Enox. 400		Enox. 400–40% Ethanol	
		10% WB	20% WB	10% WB	20% WB	10% WB	20% WB
Blood sample	Donor sex (Age)						
#1	H (56)	-	-	21.7	6.7	33.2	82.8
#2	F (32)	-	-	11.7	15.1	17.8	46.6
#3	F (32)	-	-	21.4	13.8	46	146
#4	H (34)	-	-	11	8.61	27.7	148
#5	F (30)	-	-	5.2	11.0	10.2	95.3
	mean	-	-	14.2	11.0	26.98	103.74
	SD	-	-	7.1	3.4	13.85	43.36

^(1)^ Test solutions consisted of 0.5 (10%) or 1 mL (20%) WB and 4 mL or 4.5 mL of either saline (control), Enoxaparin 400, or Enoxaparin 400–40% Ethanol solution.

-: No precipitation was observed

**Table 6:** Assessment of *in vitro* hemolysis (OD measured at 540 nm).

Blood sample	Donor sex (age)	Saline (Control)	Enox. 400	Enox. 400–40% Ethanol
#1	H (56)	0.003	0.009	0.437
#2	F (32)	0.004	0.008	0.450
#3	F (32)	0.005	0.005	0.431
#4	H (34)	0.004	0.008	0.436
#5	F (30)	0.005	0.049	0.443

## Discussion

UFH is the predominant anticoagulant lock solution used to prevent interdialytic catheter occlusion. The UFH lock concentrations used range between 1,000 and 10,000 U/mL [33]-[34]. Because of potential bleeding complications, the American Society for Diagnostic and Interventional Nephrology has recommended administering UFH at a concentration of 1,000 U/mL for locking dialysis catheters in patients without catheter occlusion [33]. UFH has no antibiofilm properties. Combining UFH with antibiotics reduces the rate of catheter infections [35]. However, the strategy is not recommended since it may promote bacterial resistance [13]. Ethanol is an antiinfectious agent with low risk of inducing bacterial resistance since it acts by protein denaturation. It could therefore be an attractive antimicrobial lock agent [36]. Its antibiofilm efficacy increases with its concentration [23].

Although catheter exposure to high ethanol concentration was suspected of reducing the elasticity of elastomers, Crnich et al [37] concluded that exposure of silicone and polyurethane catheters to a 70% ethanol lock solution does not appreciably alter their mechanical properties. In the present study, we focused on 40% ethanol solutions since this ethanol content has antibiofilm properties [23],[38] with only a marginal impact on catheter structural degradation [24], [25]. Ethanol has no anticoagulant properties and, therefore, using ethanol as a lock solution without an anticoagulant could alter catheter patency [39]. There are concerns about combining ethanol with heparin since ethanol is classically used for fractional precipitation of polysaccharides [40], [41]. Our results indicate that UFH cannot be mixed with 40% ethanol owing to incompatibility.

Although LMWHs have become the anticoagulation of choice for intermittent hemodialysis sessions in Europe [27], their use for interdialytic catheter locking has been scarcely reported [28], [29]. To the best of our knowledge, the compatibility of LMWHs/ethanol mixing solutions has not been previously documented. The optimal LMWH concentration for catheter lock remains unknown and probably differs depending on the kind of LMWH used, their pharmacodynamic properties being not clinically interchangeable [7]. A theoretical concentration of IAA for catheter locking could be estimated by extrapolating the pharmacodynamics of UFH. Thus, the recommended concentration of UFH locks represents about 10% of the daily dose of UFH currently used for venous thrombosis prophylaxis (10,000 U/day). If this 10% amount was applied to IAAs on the basis of their recommended daily dose for venous thrombosis prophylaxis [42] the resulting estimated concentrations of LMWHs for lock concentrations would approximate 200 U/mL for Enox, 280 U/mL for nadroparin and 250 U/ml for dalteparin and tinzaparin. We carried out the solubility tests on a wide range of LMWH concentrations including these estimated theoretical concentrations. The study showed that different LMWHs had different solubilities at a given ethanol content and, as generally admitted for molecules having similar structures, solubility decreased with the increase in molecular weight. Our results are in agreement with this observation: among the IAAs tested, UFH, which has the highest molecular weight, had the lowest solubility in ethanol, whereas fondaparinux, which has the lowest molecular weight, had the highest solubility. At the estimated theoretical concentrations suitable for catheter locking in 40% ethanol, tinzaparin precipitated whereas compatibility was observed for nadroparin, dalteparin and Enox. Whether these estimated theoretical LMWH concentrations could be used in a clinical setting to maintain catheter patency is unknown. Whatever the case, our study strongly suggests that if physicians decided to administer LMWH/ethanol lock solutions, they should prefer Enox, nadroparin and dalteparin over tinzaparin because the last is less soluble in ethanol.

There are few reports of anticoagulation with danaparoid and fondaparinux for circuit patency during hemodialysis [43], [44] and no data are available on their use and dose as a catheter lock solution. As calculated for LMWHs, estimated theoretical lock concentrations would approximate 150 U/mL for danaparoid and 0.25 mg/L (i.e. 232.5 U/mL) for fondaparinux. Our results indicate that danaparoid and fondaparinux at these concentrations are compatible in 40% ethanol. Among the IAAs assessed in the study, fondaparinux had the highest compatibility in ethanol.

According to the European Pharmacopeia [45], LMWH concentrations are usually determined by measuring the anti-factor X_a_ and anti-factor *II* activities in TRIS buffer and the assay is performed by absorptiometry at 405 nm. Given the various hydroalcoholic medium conditions and the non-specific low absorbance wavelengths (190–210 nm) of IAA polysaccharides, an HPLC-ELSD assay was developed. Our study suggests that visual observation of IAAs in ethanol is an accurate surrogate of stability.

As the Enox/Eth mixing solution was stable, the anti-infectious activity of such a solution was assessed using five microorganisms embedded in 24-hour old monospecies biofilms that are commonly involved in catheter infections. After 4h of exposure, significant decreases in the numbers of viable microorganisms within the biofilms were observed with 40% ethanol alone or in combination with enoxaparin. The anti-microbial activities of ethanol were not modified by the addition of enoxaparin (400 U/ml). In addition, treatment with sodium enoxaparin at 400 U/mL alone (control) did not modify the biofilm biomass whatever the microorganisms, contrary to findings reported by Shanks et al with *S*. *aureus* [8].

Depending on the microorganism studied, complete eradication of the biofilm was observed after 4 to 24h of incubation, with *K*. *pneumoniae* and *S*. *aureus* being the most resistant. In all cases, the length of time required to achieve *in vitro* biofilm eradication was shorter than the 48 to 72-hour dwell time of interdialytic locks. In a previous study using 60% ethanol treatment, we showed that a shorter incubation time (30 min) completely eradicated established biofilms formed with the same microorganisms [36]. However, such high concentrations are not compatible with the addition of injectable anticoagulant agents (IAAs) and would result in precipitation, except for sodium fondaparinux. Concentrations of ethanol between 30 and 80% have also been shown to eradicate *Candida* biofilms in a dose-dependent manner, with optimal concentration—determined as the fastest eradication with lowest ethanol strength—being 40% [38].

Exposure of catheters to ethanol is still largely debated owing to the fear of release of compounds and impairment of catheter integrity [39]. We previously described the conditions of ultra-structural integrity and chemical release of S-DC and PU-DC materials following ethanol immersion [24], [25]. S-DC material maintained integrity in ethanol at 60% [24]. For this reason, we elected to perform experiments in this study on PU-DC material.

The catheters used were made of polyurethane elastomer, whose precursors are aliphatic polycarbonates and an aliphatic isocyanate (H_12_MDI). Aliphatic polycarbonates are considered to be biocompatible and slightly toxic compounds [46]. H_12_MDI is only highly toxic when inhaled [47]. In our study, GC-MS qualitative and quantitative analyses of PU-DC migrating components in Enox/Eth immersion solution showed that the major compounds released were aliphatic polycarbonate structures with only a slight release of aliphatic H_12_MDI isomers, suggesting that PU-DC exposure to ethanol is safe.

The safety of 40% ethanol used as a lock solution and its low impact on catheter integrity observed in our study are in agreement with a recent study reporting no adverse event when locking dialysis PU-DCs were repetitively used [37].

Since ethanol can cause plasma protein precipitation at concentrations above 28% [48], we assessed plasma albumin precipitation induced by Enox/Eth. About 20% of locking solution can spill out from catheters into the blood stream during locking [49] and is replaced by WB in the catheter. Hence, we investigated plasma albumin precipitation in suspensions with ratios of 10% and 20% of WB. Albumin precipitation was observed as previously reported [48]. When the current hematocrit value (45%) and the current albumin concentration in plasma (40 g/L) were taken into account, the maximum amount of albumin precipitated in our study was lower than 0.5% of the initial albumin content of the suspension, suggesting that this precipitation has only a marginal impact in a clinical setting.

Enox/Eth has been shown to preserve anticoagulant properties [50]. This finding, in combination with our observations, strongly suggests that it could be safely used as an interdialytic lock solution in a clinical setting and be effective in preventing catheter infections and maintaining catheter patency.

We are aware that our study has some important limitations. First, the compatibility study was based on visual observations, which are less accurate than turbidimetry in measuring precipitation [51]. However, the visual observation approach is classically used to assess the solubility of antibiotics/UFH lock solutions [52] and the precipitation of protein in ethanol [48]. Second, we cannot exclude the possibility that mixing solutions defined as compatible in vitro may precipitate in vivo. Indeed, the solution locked into the patient’s catheter partly leaks into the systemic circulation. This leakage is accompanied by a concomitant blood inflow allowing plasma proteins to enter the catheter lumen and precipitate in the ethanol [48]. Third, the stability of IAAs in ethanol and the concordance between compatibility and stability were only determined with Enox400/ethanol solutions. Whether the results can be extrapolated to solutions with different Enox concentrations and to other IAAs in ethanol remains questionable. Fourth, the anti-thrombotic activity of all mixtures of Enox400 in ethanol was not assessed for all concentrations of ethanol. However, the stability of Enox400 in ethanol was shown by an HPLC-ELSD method and the absence of degradation of Enox was confirmed by the chromatographic analysis of droplets recovered at the bottom of tubes following precipitation of enoxaparin in ethanol. In addition, we recently demonstrated that Enox400 in 40% ethanol has both anti-thrombotic and anti-Xa activities [50]. With regard to the antibiofilm capacities of the Enox/Eth solution, the possibility that the eradication of biofilm embedded in silicone catheters observed after a 4-hour exposure to Enox/Eth for *S*. *epidermidis*, *P*. *aeruginosa* and *C*. *albicans* and after a 24-hour for *S*. *aureus* and *K*. *pneumoniae* was not definite since we did not assess regrowth. Another limit of our study is that polyurethane belongs to the family of elastomers, which are characterized by the heterogeneity of their components and whether the results observed after Carbothane® PU-DC exposure to Enox/Eth in our study can be extrapolated to other polyurethane catheters remains questionable. Finally, the study did not assess the mechanical properties of catheters immersed in Enox/Eth. However it has been previously shown that prolonged exposure of polyurethane and silicone catheters to 70% ethanol lock solution has a negligible impact on their mechanical properties [37]. In addition, in clinical settings, the adverse effects observed after exposure of polyurethane catheters to ethanol were only observed when ethanol content was 70% v/v or higher [39].

## Conclusions

Our study showed that, first, the compatibility of IAAs in ethanol varies depending on the kind of anticoagulant used. Second, satisfactory compatibility of enoxaparin, nadroparin, dalteparin, fondaparinux and danaparoid was observed in 40% ethanol. Of the LMWHs, enoxaparin exhibited the highest solubility and compatibility in 40% ethanol. In addition, we showed that 40% ethanol does not alter the silicone structure of catheters [24] and has only a marginal impact on polyurethane (Carbothane®) catheter structural degradation [25]. The selected Enox/Eth solution is an effective anti-biofilm solution with minor impacts on Carbothane® polymer integrity. The compounds, released after Enox/Eth or 40% ethanol exposure, were similar to those observed after prolonged saline exposure and were non-harmful. Enox/Eth may be of value as an interdialytic lock solution in preventing catheter infection. It would be timely now to perform large trials to assess the efficacy of IAAs in ethanol as interdialytic lock solutions in preventing catheter infections.

## Supporting Information

S1 Fig HPLC-ELSD profiles of enoxaparin solutions. Enoxaparin at 400 U/l diluted (A) in H_2_O, (B) In 0.9% NaCl, (C) In 50% ethanol. Enlargement of the profile from 2.5 to 5.5 min, and (D) In 70% ethanol. Enlargement of the profile from 2.5 to 5.5 min.
(DOCX)

S1 Table Testing grid for the visual determination of unfractionated heparin precipitation in ethanol. Influence of relative concentrations, time of contact and temperature on UFH solubility.
(DOCX)

S2 Table Testing grid for the visual determination of enoxaparin 400 U/mL precipitation in ethanol. Influence of relative ethanol contents, time of contact and temperature on enoxaparin 400 U/mL solubility.
(DOCX)

S3 Table Testing grid for the visual determination of danaparoid precipitation in ethanol. Influence of relative concentrations, time of contact and temperature on danaparoid solubility.
(DOCX)

S4 Table Testing grid for the visual determination of fondaparinux precipitation in ethanol. Influence of relative concentrations, time of contact and temperature on fondaparinux solubility.
(DOCX)

S5 Table Ethanol content in enoxaparin 400U/mL/ethanol at different levels of ethanol content (40%, 45%, and 50%). (DOCX)

S1 Text Description of the HPLC-ELSD method used for enoxaparin concentration measurement. (DOCX)
