# Supplementary material for: Compatibility of Injectable Anticoagulant Agents in Ethanol; In Vitro Antibiofilm Activity and Impact on Polyurethane Catheters of Enoxaparin 400 U/mL in 40% v/v Ethanol
Source: PLoS One. 2016 Jul 21;11(7):e0159475. doi: 10.1371/journal.pone.0159475 (PMC4956118; doi:10.1371/journal.pone.0159475)
Supplement: S1 Fig — Enoxaparin at 400 U/l diluted (A) in H2O, (B) In 0.9% NaCl, (C) In 50% ethanol. Enlargement of the profile from 2.5 to 5.5 min, and (D) In 70% ethanol. Enlargement of the profile from 2.5 to 5.5 min. (DOCX) [file pone.0159475.s001.docx]

S1 Figure. HPLC-ELSD profiles of enoxaparin solutions

Enoxaparin at 400 U/l diluted (A) in H_2_O, (B) In 0.9% NaCl, (C) In 50% ethanol. Enlargement of the profile from 2.5 to 5.5 min, and (D) In 70% ethanol. Enlargement of the profile from 2.5 to 5.5 min
