# Supplementary material for: Compatibility of Injectable Anticoagulant Agents in Ethanol; In Vitro Antibiofilm Activity and Impact on Polyurethane Catheters of Enoxaparin 400 U/mL in 40% v/v Ethanol
Source: PLoS One. 2016 Jul 21;11(7):e0159475. doi: 10.1371/journal.pone.0159475 (PMC4956118; doi:10.1371/journal.pone.0159475)
Supplement: S1 Table — Influence of relative concentrations, time of contact and temperature on UFH solubility. (DOCX) [file pone.0159475.s002.docx]

S1 Table. Testing grid for the visual determination of unfractionated heparin precipitation in ethanol. Influence of relative concentrations, time of contact and temperature on UFH solubility.

| Ethanol  (%, v/v) | UFH  (U/mL) | Room temperature  1min 1h 24h 48h 72h | | | | | 37°C  1min 1h 24h 48h 72h | | | | |
| --- | --- | --- | --- | --- | --- | --- | --- | --- | --- | --- | --- |
| 30 | 50 | 0 | 0 | 0 | 0 | 0 | 0 | 0 | 0 | 0 | 0 |
|  | 100 | 0 | 0 | 0 | 0 | 0 | 0 | 0 | 0 | 0 | 0 |
|  | 200 | 0 | 0 | 0 | 0 | 0 | 0 | 0 | 0 | 0 | 0 |
|  | 300 | 0 | 0 | 0 | 0 | 0 | 0 | 0 | 0 | 0 | 0 |
|  | 400 | 0 | 0 | 0 | 0 | 0 | 0 | 0 | 0 | 0 | 0 |
|  | 500 | 0 | 0 | 0 | 0 | 0 | 0 | 0 | 0 | 0 | 0 |
|  | 800 | 0 | 0 | 0 | 0 | 0 | 0 | 0 | 0 | 0 | 0 |
|  | 1000 | 0 | 0 | 0 | 0 | 0 | 0 | 0 | 0 | 0 | 0 |
|  | 1200 | 0 | 0 | 0 | 0 | 0 | 0 | 0 | 0 | 0 | 0 |
|  | 1500 | 0 | 0 | 0 | 0 | 0 | 0 | 0 | 0 | 0 | 0 |
|  | 2500 | 0 | 0 | 0 | 0 | 0 | 0 | 0 | 0 | 0 | 0 |
| 35 | 50 | 0 | 0 | 0 | 0 | 0 | 0 | 0 | 0 | 0 | 0 |
|  | 100 | 0 | 0 | 0 | 0 | 0 | 0 | 0 | 0 | 0 | 0 |
|  | 200 | 0 | 0 | 0 | 0 | + | 0 | 0 | 0 | 0 | 0 |
|  | 300 | 0 | 0 | 0 | + | + | 0 | 0 | 0 | 0 | 0 |
|  | 400 | 0 | 0 | + | + | + | 0 | 0 | 0 | 0 | 0 |
|  | 500 | 0 | 0 | + | + | + | 0 | 0 | 0 | 0 | 0 |
|  | 800 | 0 | + | + | + | + | 0 | 0 | 0 | 0 | 0 |
|  | 1000 | + | + | + | + | + | 0 | 0 | 0 | 0 | 0 |
|  | 50 | + | + | + | + | + | 0 | 0 | 0 | 0 | 0 |
|  | 100 | + | + | + | + | + | 0 | 0 | 0 | 0 | 0 |
|  | 200 | + | + | + | + | + | + | + | 0 | 0 | 0 |
|  | 300 | + | + | + | + | + | + | + | 0 | 0 | 0 |
| 40 | 400 | + | + | + | + | + | + | + | 0 | 0 | 0 |
|  | 500 | + | + | + | + | + | + | + | + | + | + |
|  | 800 | + | + | + | + | + | + | + | + | + | + |
|  | 1000 | + | + | + | + | + | + | + | + | + | + |
| 45 | 300 | + | + | + | + | + | + | + | + | + | + |
|  | 100 | + | + | + | + | + | + | + | + | + | + |
|  | 200 | + | + | + | + | + | + | + | + | + | + |
|  | 300 | + | + | + | + | + | + | + | + | + | + |
|  | 400 | + | + | + | + | + | + | + | + | + | + |

0, absence of precipitates including cloudiness, film deposit and droplets

+, presence of precipitates including cloudiness, film deposit and droplets
