# Supplementary material for: Compatibility of Injectable Anticoagulant Agents in Ethanol; In Vitro Antibiofilm Activity and Impact on Polyurethane Catheters of Enoxaparin 400 U/mL in 40% v/v Ethanol
Source: PLoS One. 2016 Jul 21;11(7):e0159475. doi: 10.1371/journal.pone.0159475 (PMC4956118; doi:10.1371/journal.pone.0159475)
Supplement: S2 Table — Influence of relative ethanol contents, time of contact and temperature on enoxaparin 400 U/mL solubility. (DOCX) [file pone.0159475.s003.docx]

S2 Table. Testing grid for the visual determination of enoxaparin 400 U/mL precipitation in ethanol. Influence of relative ethanol contents, time of contact and temperature on enoxaparin 400 U/mL solubility.

| Ethanol  (%, v/v) | at Room temperature | | | | | at 37°C | | | | |
| --- | --- | --- | --- | --- | --- | --- | --- | --- | --- | --- |
|  | 1min | 1h | 24h | 48h | 72h | 1min | 1h | 24h | 48h | 72h |
| 30 | 0 | 0 | 0 | 0 | 0 | 0 | 0 | 0 | 0 | 0 |
| 40 | 0 | 0 | 0 | 0 | 0 | 0 | 0 | 0 | 0 | 0 |
| 45 | + | + | + | + | + | 0 | 0 | 0 | 0 | 0 |
| 50 | + | + | + | + | + | + | + | + | + | + |
| 60 | + | + | + | + | + | + | + | + | + | + |
| 70 | + | + | + | + | + | + | + | + | + | + |

0, absence of precipitates including cloudiness, film deposit and droplets

+, presence of precipitates including cloudiness, film deposit and droplets
