# Supplementary material for: Compatibility of Injectable Anticoagulant Agents in Ethanol; In Vitro Antibiofilm Activity and Impact on Polyurethane Catheters of Enoxaparin 400 U/mL in 40% v/v Ethanol
Source: PLoS One. 2016 Jul 21;11(7):e0159475. doi: 10.1371/journal.pone.0159475 (PMC4956118; doi:10.1371/journal.pone.0159475)
Supplement: S5 Table — (DOCX) [file pone.0159475.s006.docx]

S5 Table. Ethanol content in enoxaparin 400U/mL/ethanol at different levels of ethanol content (40%, 45%, and 50%).

| Theoretical ethanol content in Enox400/ethanol | 40 % | 45% | 50% |
| --- | --- | --- | --- |
| Calculated ethanol mean content in % (v/v) | 40.5 ± 0,2  (n=6) | 44.9 ± 0.4  (n=3) | 49.8 ± 0,4  (n=3) |
| CV (%) | 0.5 | 0.9 | 0.8 |
| Recovery (%) | 101.2 **±** 0.5 | 99.8 **±** 0.9 | 99.6 **±** 0.8 |

Enox400, enoxaparin at a fixed concentration of 400 U/mL
