# Supplementary material for: Compatibility of Injectable Anticoagulant Agents in Ethanol; In Vitro Antibiofilm Activity and Impact on Polyurethane Catheters of Enoxaparin 400 U/mL in 40% v/v Ethanol
Source: PLoS One. 2016 Jul 21;11(7):e0159475. doi: 10.1371/journal.pone.0159475 (PMC4956118; doi:10.1371/journal.pone.0159475)
Supplement: S1 Text — (DOCX) [file pone.0159475.s007.docx]

S1 text. Description of the HPLC-ELSD method used for enoxaparin concentration measurement

HPLC separation was performed with an Agilent 1200 liquid chromatography system (Agilent Technologies, Palo Alto, CA, USA) combined with an evaporative light scattering detector (ELSD) and using an Agilent Eclipse XD8-C18 column (150 mm x 4.6 mm i.d., 5µ pore size) kept at room temperature. The mobile phase was methanol/distilled water (50/50, v/v) and the separation was obtained in isocratic mode at a flow rate of 0.3 mL/min. Injection volume was 20 µL. The ELSD was connected to the chromatograph with an evaporator temperature at 40°C; the nebulizing gas was N_2_ at 3.5 bar and the gain (PM) was maintained at 3.0.

Analyses were performed in triplicate following adequate 1/8 dilution of samples in serum saline, and calibration curves were established using six final levels of enoxaparin concentration (range 25 to 120 U/mL) prepared in serum saline. For samples presenting a precipitate or cloudiness or droplets, a preliminary centrifugation at 1600 g for 5 minutes was done and the enoxaparin assay was carried out on the clear supernatant. The equation log A = a log C + log b, where A is the area of the chromatographic peak, C the concentration of enoxaparin, b the response factor and a the response index measured from the slope of the curve, was used to characterize the law of the quantitative response of the detector as described by S. Heron*. The mean curve of calibration allowed the determination of the parameters a (1.775) and b (0.048) and the regression coefficient was R^2^ = 0.993. A blank sample (40% ethanol in 0.9 % sodium chloride) was analysed to confirm the absence of interference.

*Heron S, Malooumbi MG, Dreux M, Verette E, Tchapla A. *J Chromatogr* 1161(1-2): 152-156, 2007
